# Genomic Insights into Adaptation of *Lagerstroemia suprareticulata* to Limestone Karst Habitats

**DOI:** 10.3390/plants15040629

**Published:** 2026-02-16

**Authors:** Shuo Zhang, Yi Li, Ying Xie, Xiaomei Deng, Ye Sun

**Affiliations:** Guangdong Key Laboratory for Innovative Development and Utilization of Forest Plant Germplasm, College of Forestry and Landscape Architecture, South China Agricultural University, Guangzhou 510642, China; szhang0808@163.com (S.Z.); lliyi2025215@163.com (Y.L.); xying233943@163.com (Y.X.)

**Keywords:** endangered plants, genetic variation, limestone karst, *Lagerstroemia suprareticulata*, whole-genome resequencing

## Abstract

*Lagerstroemia suprareticulata*, an endemic ornamental species in limestone karst ecosystems of Guangxi—a global biodiversity hotspot—holds significant ecological value. However, habitat degradation and anthropogenic pressures have driven this species to the brink of extinction, leading to its classification as Endangered (EN) on the China Biodiversity Red List. To address this crisis, we conducted whole-genome resequencing to generate single-nucleotide polymorphisms (SNPs) for comprehensive analyses of genetic diversity, population structure, demographic history, and adaptive variation. Our results reveal four distinct genetic clusters in *L. suprareticulata*, all of which share a history of population expansion followed by contraction. Maximum entropy modeling (MaxEnt) projects a severe contraction in the range under high-carbon-emission scenarios. Selective sweep analysis identified genomic regions under positive selection, including those associated with protein homeostasis, metabolism, signal transduction, and developmental regulation. Genotype–environment association (GEA) analysis further identified adaptive SNPs linked to temperature and precipitation, which were enriched in genes regulating transmembrane transport, stress response, and the immune system. Additionally, risk of non-adaptedness (RONA) analysis identified high-risk populations. By integrating genomic data with advanced analytical approaches, this study enhances our understanding of the adaptive mechanisms of *L. suprareticulata* to limestone karst habitats and provides critical insights for its conservation.

## 1. Introduction

Karst landforms, a unique global landform type primarily composed of soluble rock formations, are particularly prominent in southwest China, where they are recognized as terrestrial biodiversity hotspots [1]. The alkaline soils resulting from limestone dissolution in these regions foster specialized plant habitats. Endemic species have evolved distinct physiological and ecological adaptations to thrive in the arid, highly alkaline, and shallow-soil conditions of karst environments [2,3]. Water scarcity in karst regions necessitates drought adaptation, with plants employing strategies such as leaf physiology adjustments, root system expansion, organ morphology changes, and hormonal signaling regulation [4]. Additionally, the high calcium content of karst soils has spurred research into related adaptive mechanisms through experiments and various omics technologies. For instance, *Camellia limonia* shows increased chlorophyll content and leaf area under calcium stress, with flavonoid biosynthesis identified as a key regulatory pathway in its adaptive response [5]. Comparative studies of *Urophysa* and its non-karst counterparts highlight enhanced stress response, calcium signaling, and water retention [6]. Furthermore, the calcium channel gene *TPC1* has been implicated in adaptation to high-calcium soils [7,8]. While these studies provide valuable insights, further research is urgently needed to fully understand karst plant adaptations.

Rapid climate change poses significant threats to plant species, exacerbating the risk of extinction [9]. Within this context, narrow-endemic species, particularly those adapted to karst ecosystems, warrant heightened conservation priority due to their unique vulnerabilities [10]. These plants exhibit specialized adaptations, such as tolerance for drought and alkaline soils, but their fragile ecosystems face escalating threats from climate change and human activities [11,12]. Comprehensive surveys and scientific research are critical for identifying and addressing the multifaceted challenges faced by these endangered species [13,14]. Consequently, robust methodologies for assessing survival status and threat patterns are essential for developing effective conservation strategies in limestone karst ecosystems.

Advancements in high-throughput sequencing technologies have significantly expanded the application of population genomics in endangered species [15]. This approach allows for the analysis of population structure, effective population size, demographic history, and the identification of adaptive genetic variation [16,17]. While population genomics effectively identifies genomic regions of elevated differentiation, a key focus in adaptive gene searches based solely on sequence data [18], this approach’s exclusive reliance on genetic metrics frequently neglects the environmental drivers of adaptive divergence. To address this limitation, landscape genomics integrates environmental data with genomic information to identify adaptive loci associated with specific ecological conditions, thereby revealing the genomic mechanisms underlying species adaptation to environmental variability and enhancing understanding of their evolutionary resilience [19]. By combining population genomics and landscape genomics, researchers can comprehensively assess the conservation status of endangered plant species, supporting informed conservation decisions [20]. Furthermore, species distribution models (SDMs), which relate species occurrence data and environmental variables to predict distribution patterns, can improve conservation planning by forecasting future suitable habitats [21].

The genus *Lagerstroemia*, renowned for spectacular flowers and diverse colors, encompasses several horticulturally significant species, many of which also exhibit notable medicinal properties. Widely cultivated in gardens, streetscapes, and parks, these plants are also popular choices for bonsai artistry [22]. Contemporary research on *Lagerstroemia* primarily focuses on genetic breeding [23], phylogenetics [24,25,26], and medicinal applications [27,28], yielding substantial scientific advancements. Although genetic breeding programs have conserved genetic resources of this genus, conservation efforts for wild endangered species remain inadequate. *Lagerstroemia suprareticulata*, a small tree or shrub distinguished by white petals and leaves with prominent reticulate venation on the adaxial surface [29], represents both a highly prized ornamental specimen and a vital germplasm resource within the genus. This species is endemic to limestone mountainous habitats in Guangxi Zhuang Autonomous Region. Despite its strong adaptability to karst ecosystems, natural populations face severe threats from illegal logging, climate change, and habitat fragmentation [30]. Currently classified as Endangered (EN) according to the China Biodiversity Red List (http://protection.especies.cn/redlist/list, accessed on 1 March 2025), *L. suprareticulata* requires urgent conservation interventions [31]. Nevertheless, fundamental aspects of population structure, genetic diversity, and adaptive mechanisms remain largely unexplored in this genus. To address these gaps, we employed whole-genome resequencing combined with multiple advanced analytical approaches to elucidate these critical characteristics, thereby informing effective conservation strategies.

This study employs single-nucleotide polymorphisms (SNPs) from whole-genome resequencing to investigate genetic diversity, population structure, and genomic signatures of adaptation to limestone karst habitats in *L. suprareticulata*. Our research focuses on four key objectives: (1) characterizing the population structure and genetic diversity of *L. suprareticulata*; (2) reconstructing its demographic history to infer evolutionary trajectories; (3) predicting potential suitable habitats and assessing future adaptability through species distribution modeling; and (4) uncovering the genomic basis of its adaptation to limestone karst ecosystems. By integrating these analyses, we develop a comprehensive genomic framework to inform conservation strategies, addressing critical gaps in understanding the species resilience to threats such as illegal logging, habitat fragmentation and climate change. This work advances biodiversity conservation in limestone karst regions, highlighting the urgent need to protect *L. suprareticulata* amid ongoing climate change.

## 2. Results

### 2.1. SNP Calling

Whole-genome resequencing generated 169.89 Gb of data with an average sequencing depth of 12.51×. After alignment and duplicate removal, 33,458,037 SNPs were initially identified. Following filtering for minor allele frequency (MAF) and linkage disequilibrium (LD), 2,392,230 high-quality SNPs remained for subsequent studies.

### 2.2. Population Structure

**Figure 1:**
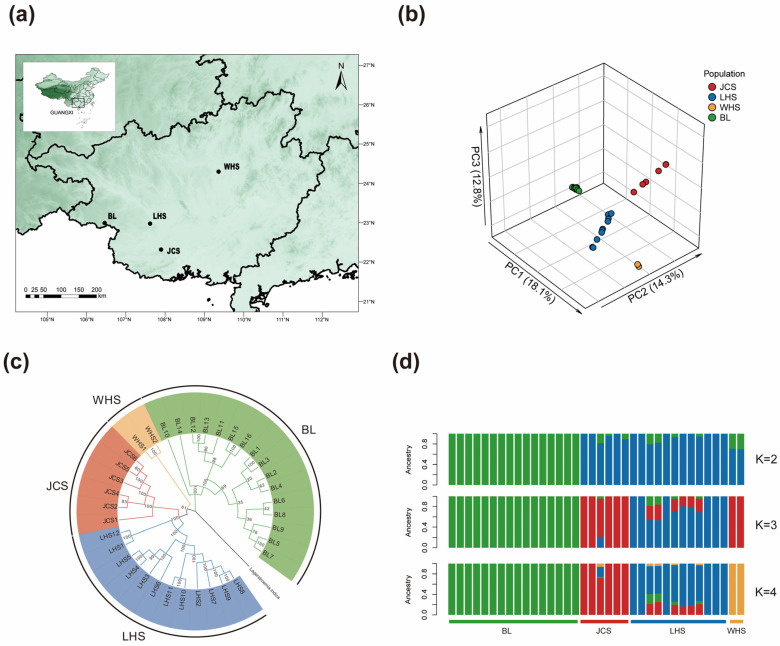
Population structure of *Lagerstroemia suprareticulata*. (**a**) Geographic distribution of sampling locations (black dots). (**b**) Principal component analysis (PCA) plot (PC1-PC3: 18.1%, 14.3%, and 12.8% of the total variance, respectively). (**c**) Neighbor-joining (NJ) tree of 36 *L. suprareticulata* individuals (branches colored by population). (**d**) ADMIXTURE results (K = 2–4, bars represent individuals, with colors indicating ancestry proportions).

Principal component analysis (PCA) revealed four distinct genetic clusters in *L. suprareticulata*, each corresponding precisely to one of four geographic populations (Figure 1a). The first three principal components (PC1–PC3) accounted for 18.1%, 14.3%, and 12.8% of the total genetic variance, respectively (Figure 1b). Consistent results were obtained from the neighbor-joining (NJ) tree, with four monophyletic groups corresponding to the four geographic populations (Figure 1c). ADMIXTURE analysis revealed minimal cross-validation (CV) error at K = 1, with error increasing as K values rose. At K = 2, the BL population diverged from the remaining three populations, and by K = 4, all four populations exhibited distinct genetic profiles, consistent with PCA and NJ results (Figure 1d). These analyses clearly demonstrate genetic differentiation among *L. suprareticulata* populations, with four ancestral components representing the optimal population structure.

The nucleotide diversity (π) across the four *L. suprareticulata* populations ranged from 0.001779 to 0.002137, indicating low overall genetic variability (Table 1). Among these populations, LHS exhibited the highest π value, while WHS showed the lowest. Pairwise population differentiation index (F_ST_) values ranged from 0.0409 to 0.0678 (Table S1), with the most pronounced differentiation observed between LHS and WHS and the least between JCS and LHS. These findings suggest moderate genetic differentiation among the populations. Tajima’s D values for all four populations were slightly positive, indicating historical population bottlenecks (Table 1). The WHS population exhibited a significantly elevated Tajima’s D value compared to the other three populations, suggesting that it may have experienced a more pronounced bottleneck.

**Figure 2:**
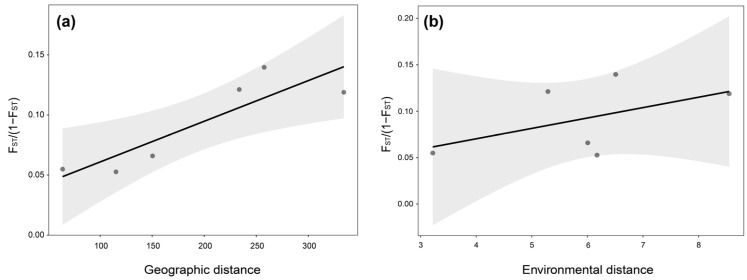
Mantel test results evaluating (**a**) isolation-by-distance (IBD) and (**b**) isolation-by-environment (IBE) by comparing genetic, geographic, and environmental distance matrices. A significant IBD pattern was detected (Mantel’s r = 0.77, *p* = 0.021), whereas no significant IBE pattern was observed.

The Mantel test results revealed distinct relationships between genetic differentiation and environmental versus geographic variables in *L. suprareticulata*. A significant positive correlation (*p* = 0.021) was observed between genetic distance and geographic distance across the four populations (Figure 2a), indicating that spatial isolation plays a key role in shaping genetic divergence. In contrast, the correlation between genetic distance and environmental distance was weak and nonsignificant (*p* = 0.309, Figure 2b). These findings suggest that geographic isolation is the primary driver of population differentiation in this species.

### 2.3. Historical Demography

**Figure 3:**
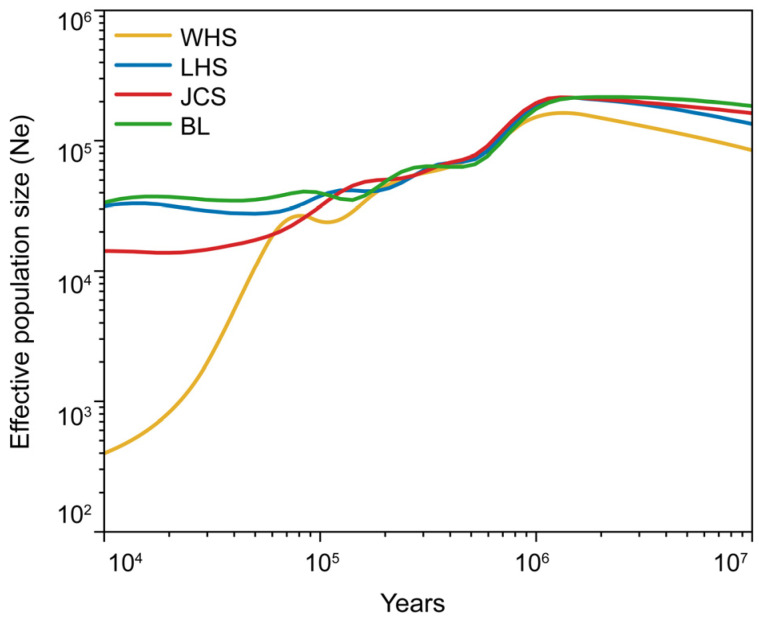
Historical trajectories of effective population size (Ne) in *Lagerstroemia suprareticulata* populations inferred by SMC++.

Using SMC++, we inferred effective population sizes (Ne) of *L. suprareticulata* populations in the limestone karst regions of Guangxi, revealing a shared expansion–contraction history across all four populations (Figure 3). Following a prolonged expansion phase lasting until ~1 million years ago (Mya), *L. suprareticulata* experienced a population bottleneck, triggering a demographic decline. Although two brief recovery events occurred around 0.5 Mya and 0.1 Mya, these fluctuations were insufficient to reverse the long-term contraction trend. By 0.01 Mya, three populations maintained a Ne of nearly 20,000 individuals, while the WHS population sharply declined to approximately 500 individuals.

### 2.4. Suitable Habitat Distribution

The MaxEnt model was employed to predict habitat suitability for *L. suprareticulata* across past, present, and future time periods. Environmental variable contributions were assessed using the Jackknife test, with temperature and soil properties identified as primary drivers. Among these, six variables emerged as the most influential: bio1 (Annual Mean Temperature), awc_class (Soil Available Water Content), t_clay (Clay content), bio8 (Mean Temperature of Driest Quarter), bio6 (Min Temperature of Coldest Month), and bio10 (Mean Temperature of Warmest Quarter). Collectively, these variables accounted for 88.2% of the total contribution to habitat suitability (Table 2). Notably, bio6 and awc_class contributed the highest percentages (38.6% and 22.3%, respectively), with their combined contribution exceeding 60%. Bio1 contributed 14.5% and showed a permutation importance of 28.3%, underscoring its significant role in determining species distribution limits.

Based on the Jackknife test and Pearson correlation analysis, four bioclimatic variables (bio1, bio6, bio9, bio10) and three soil variables (awc_class, t_clay, s_ph_h2o) were identified as the most influential for inclusion in MaxEnt modeling (Figures S2 and S3). The average area under the receiver operating characteristic curve (AUC) across 10 MaxEnt runs was 0.996, indicating exceptional model performance and high predictive accuracy.

MaxEnt modeling results indicate that the current suitable habitat for *L. suprareticulata* is primarily concentrated in western Guangxi, with additional moderately to lowly suitable areas distributed across Guangdong, Hainan, and Taiwan (Figure S3). The total contemporary suitable habitat spans 96,127.78 km^2^, of which 11,613.89 km^2^ is classified as highly suitable. Compared to historical periods, the suitable habitat has experienced significant contraction, declining from the Last Glacial Maximum (LGM; 141.35% of current area) and Mid-Holocene (MH; 172.22% of current area) to the present day (Table 3, Figure S3).

**Figure 4:**
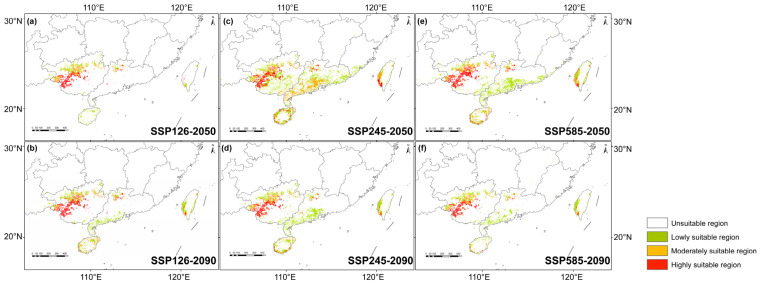
Projected potential suitable habitat for *Lagerstroemia suprareticulata* under future climate scenarios: SSP126 (**a**,**b**), SSP245 (**c**,**d**), and SSP585 (**e**,**f**) for mid-century (2050) and end-of-century (2090).

Under the low-emission SSP126 scenario, the highly suitable habitat for *L. suprareticulata* is projected to expand gradually, reaching 108.23% of the current baseline by 2050 and 141.82% by 2090. In contrast, under SSP245, the highly suitable habitat rises to 160.14% by 2050 before declining to 124.31% by 2090, while under SSP585 it increases to 177.87% in 2050 and then drops to 111.81% in 2090 (Figure 4). Furthermore, the remaining suitable habitat will become increasingly fragmented and restricted to areas near Baise, Chongzuo and Hechi City in western Guangxi. Large portions of moderately or lowly suitable habitats will disappear, leading to an overall decline in total suitable habitat. These findings indicate that under high-carbon-emission conditions, the long-term survival of *L. suprareticulata* in lowly or moderately suitable habitats will be threatened. Although the highly suitable area may expand slightly, it could also become more fragmented, warranting attention in conservation planning.

### 2.5. Selective Sweep Detection

**Figure 5:**
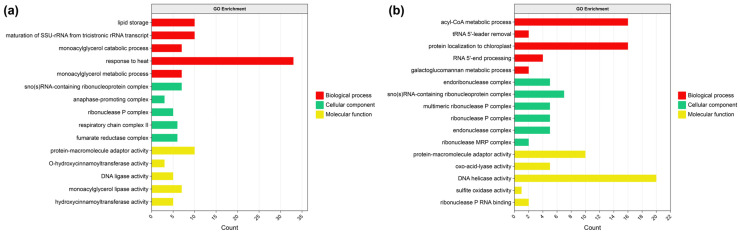
Gene Ontology (GO) enrichment analysis of adaptive loci. (**a**) Selective sweep loci; (**b**) genotype–environment associated loci. Bar lengths indicate the number of enriched genes associated with the top 5 GO terms (ranked by FDR-corrected *p*-value) across three ontology categories: biological process, cellular component, and molecular function.

Selective sweep analysis identified 8625 regions under positive selection. Gene Ontology (GO) enrichment analysis revealed that these regions encompass genes associated with cellular components, biological processes, and molecular functions (Figure 5a, Table S3). Within cellular components, enriched GO terms included the anaphase-promoting complex, ribonucleoprotein complex, fumarate reductase complex, respiratory chain complex II, and multimeric ribonuclease P complex. For biological processes, enriched terms comprised monoacylglycerol metabolic process, response to heat, lipid storage, monoacylglycerol catabolic process, and maturation of SSU-rRNA from tricistronic rRNA transcript. Regarding molecular functions, enriched activities included monoacylglycerol lipase activity, protein-macromolecule adaptor activity, DNA ligase activity, and O-hydroxycinnamoyl transferase activity.

**Figure 6:**
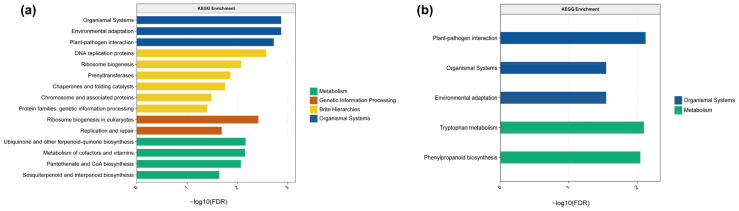
KEGG enrichment analysis of adaptive loci. (**a**) Selective sweep loci; (**b**) genotype–environment associated loci. Bar length represents the −log10 (FDR) value, reflecting the significance of enrichment. Color code denotes KEGG pathway categories: Metabolism, Genetic Information Processing, Organismal Systems, and Brite Hierarchies.

These positively selected genes were enriched in five major Kyoto Encyclopedia of Genes and Genomes (KEGG) pathway categories: (1) Cellular processes (including structural proteins and organismal systems), (2) Stress response (encompassing environmental adaptation, plant–pathogen interaction, and chaperone/folding catalysis), (3) Metabolism and biosynthesis (involving cofactors/vitamin metabolism, ubiquinone/terpenoid-quinone biosynthesis, pantothenate/CoA biosynthesis, and sesquiterpenoid/diterpenoid biosynthesis), (4) Genetic information processing (including replication/repair, chromosome-associated proteins, and eukaryotic ribosome biogenesis), and (5) Protein families (linked to DNA replication and genetic information processing) (Figure 6a, Table S4). Key genes were identified through The Basic Local Alignment Search Tool (BLAST) v2.16.0 alignments against the Arabidopsis gene database and classified into three functional categories: (1) Stress response and protein homeostasis (e.g., *HSP101*, *CLPB3*, *ABCI13*, *XI-I*), (2) Metabolism and biosynthesis (e.g., *PDS3*, *CYSC1*, *RPS5A*, *PAI1*, *HCT*), and (3) Signal transduction and developmental regulation (e.g., *TOPP4*, *eIF3-7*, *AT4G28450*).

### 2.6. Genotype–Environment Association (GEA) Analysis

Using the gradient forest (GF) model, we analyzed 19 temperature and precipitation variables and identified bio1 (Annual Mean Temperature), bio9 (Mean Temperature of Driest Quarter), bio16 (Precipitation of Wettest Quarter), bio12 (Annual Precipitation), bio8 (Mean Temperature of Wettest Quarter), and bio14 (Precipitation of Driest Month) as the most critical environmental factors influencing the adaptability of *L. suprareticulata* (Figures S4 and S5). After addressing multicollinearity with reference to Pearson correlation and GF analyses, bio1 (Annual Mean Temperature), bio2 (Mean Diurnal Range), bio6 (Minimum Temperature of Coldest Month), bio12 (Annual Precipitation), and bio16 (Precipitation of Wettest Quarter) were selected for adaptive loci screening.

Latent Factor Mixed Models (LFMM) analysis identified 37,443 adaptive SNPs, whereas Redundancy analysis (RDA) analysis detected 50,198. The overlap between the two methods revealed 15,579 high-confidence adaptive SNPs. Subsequent GO enrichment analysis uncovered significant associations with genes involved in cellular components, biological processes, and molecular functions (Figure 5b, Table S5). The enriched biological processes included acyl-CoA metabolism, protein localization to chloroplast, RNA 5′-end processing, tRNA 5′-leader removal, and galactoglucomannan metabolic process. For cellular components, enriched terms encompassed RNA-containing ribonucleoprotein complex, endonuclease complex, ribonuclease P complex (including multimeric forms), and endoribonuclease complex. In terms of molecular functions, enrichments were observed in DNA helicase activity, ribonuclease P RNA binding, protein–macromolecule adaptor activity, oxo-acid-lyase activity, and sulfite oxidase activity. KEGG pathway analysis revealed significant enrichment in the following pathways: enzymes with EC numbers, plant–pathogen interaction, tryptophan metabolism, phenylpropanoid biosynthesis, organismal systems, and environmental adaptation (Figure 6b, Table S6).

Subsequently, we performed BLAST searches against the *A. thaliana* database to identify key genes implicated in *L. suprareticulata*’s adaptation to limestone karst environments. These genes enhance stress resistance through diverse regulatory and repair mechanisms, including protein modification and degradation (e.g., *UBC1*, *UBC2*, *UBC35*, *CLPB3*, *UBL*); RNA processing and splicing (e.g., *LSM2*, *SCL33*, *SUS2*, *YLS8*, *FIB2*), vesicle transport (e.g., *AT1G62020*, *SNF7.2*, *GOS12*, *RABE1c*, *VAMP727*, *ABCB25*), secondary metabolism (e.g., *PAL1*, *CYP98A3*, *CAD5*, *LDOX*), cell structure maintenance (e.g., *GUT2*, *GUT1*, *CDC2*, *APC10*, *FTSZ1-1*), and DNA repair (e.g., *UVH6*, *RFC2*, *PRL*, *CDC2*, *RHL2*).

### 2.7. Risk of Non-Adaptation (RONA) Analysis

**Figure 7:**
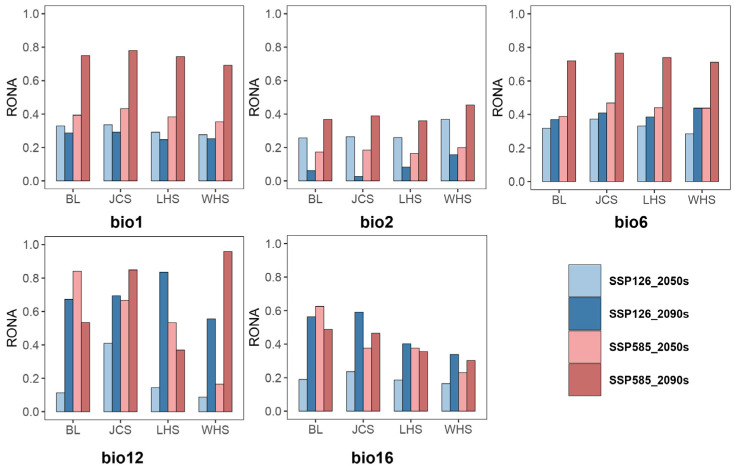
Risk of Non-Adaptation (RONA) values for *Lagerstroemia suprareticulata* populations, encompassing six bioclimatic variables and two Shared Socioeconomic Pathways (SSPs). Blue bars represent low-emission SSP126 scenarios, whereas red bars indicate high-emission SSP585 scenarios. The bar length is proportional to the RONA value, with longer bars signifying greater exposure to climate risk.

We employed the Risk of Non-Adaptedness (RONA) metric to evaluate the population-level vulnerability of *L. suprareticulata* to six bioclimatic variables, for both near-term (2050) and long-term (2090) climate projections (Figure 7). Higher RONA values signify greater exposure to climate risk. Under bio1 and bio6, all four populations showed comparable RONA values, indicating uniform vulnerability. In contrast, bio2 disproportionately affected the WHS population, which exhibited elevated RONA values. For bio16, the BL, JCS, and LHS populations demonstrated greater vulnerability than WHS, and this pattern was also observed for bio12, except under the SSP585-2090 scenario.

## 3. Discussion

PCA and NJ tree analyses clearly resolved *L. suprareticulata* into four distinct populations. Although the cv error was minimal at K = 1, a pronounced population structure emerged at K = 4, supporting the PCA and NJ Tree results. These results collectively indicate that *L. suprareticulata* populations are structured into genetically distinct groups. The BL population, distributed within the Bangliang Nature Reserve (486–926 m elevation), inhabits steep limestone karst habitats [32]. Its higher-elevation distribution likely facilitated earlier historical divergence than that of the other three populations [33]. The regional limestone karst topography acts as a natural barrier to gene flow, thereby promoting population isolation [34,35]. The remaining three populations, geographically separated by mountain ranges and substantial distances, have gradually developed genetic differentiation over time. Additionally, human-induced habitat degradation and fragmentation have further restrict gene flow among these populations [12]. *L. suprareticulata* exhibits low genetic diversity (π = 0.0021), consistent with its endangered status—a pattern observed in other threatened plant species [36,37]. Pairwise F_ST_ values reveal low-to-moderate levels of genetic differentiation among populations. Mantel test results confirmed significant geographic isolation as a key driver of population differentiation in this limestone karst species [38]. This aligns with studies of other karst-endemic plants, such as *Heteroplexis* species, in which strong geographic isolation has led to pronounced population structure [39].

All four *L. suprareticulata* populations exhibited an expansion–contraction demographic history, consistent with patterns observed in other *Lagerstroemia* species [40]. Their Ne initially expanded, peaking around 1 Mya, likely benefiting from the relatively warmer climate of the Miocene and Pliocene [41,42]. Additionally, the extensive karstification phase during the Miocene in Guangxi may have provided a more favorable environment for *L. suprareticulata*, enabling its Ne to maintain a sustained upward trend [35,43]. This expansion was followed by a prolonged decline, with several minor rebounds failing to reverse the overall trend. A slight Ne increase at 0.1 Mya may be linked to the emergence of a wetter climate in Guangxi [44], as increased precipitation could have improved survival conditions. Quaternary glacial–interglacial climate fluctuations profoundly impacted plant survival, driving repeated cycles of population expansions and contractions across species [45]. The WHS population experienced a particularly sharp decline around 0.01 Mya, likely exacerbated by its geographic isolation within Liuzhou city [39]. Tajima’s D values for all four populations were slightly positive, indicating that they experienced historical bottlenecks. Notably, the WHS population exhibited significantly higher Tajima’s D than the other three populations, suggesting it may have undergone a more severe bottleneck. These findings align with the population history dynamics inferred by SMC++. Historical dynamics show no signs of recovery, and ongoing climate fluctuations and habitat fragmentation in the limestone karst ecosystem pose further risks of decline. Given the projected reduction in highly suitable habitats in this region, conservation efforts should prioritize safeguarding marginal populations, including the WHS population. To boost Ne recovery and promote genetic diversity, assisted gene flow could be implemented as a potential intervention [46].

MaxEnt, a widely used species distribution model (SDM), is particularly effective for modeling the potential habitat of endangered species with sparse occurrence data [47,48]. This approach is essential for informing biodiversity conservation strategies and management planning [49]. For *L. suprareticulata*, the variables bio1, bio6, and awc_class were identified as key predictors, underscoring its sensitivity to thermal regimes and soil moisture availability. This moisture dependence is particularly critical in limestone karst ecosystems, where water scarcity frequently occurs [50,51]. Our MaxEnt modeling consistently identified Guangxi as the optimal habitat for *L. suprareticulata*, a finding confirmed by field surveys. While marginal suitable habitats were detected in Guangdong, Hainan, and Taiwan, these regions exhibited consistently low habitat suitability scores, suggesting limited ecological viability for population establishment. The suitable habitat of this species has experienced a substantial reduction from both the LGM and MH periods to the present day. During the LGM, colder climates restricted suitable habitats to a limited range [52,53], whereas the warmer, more humid conditions of the MH period facilitated habitat expansion [54]. Notably, some high-suitability areas identified by MaxEnt appear to have historically supported *L. suprareticulata*. However, our current surveys detected no extant populations in these regions—likely due to anthropogenic pressures, including illegal harvesting and excavation [30]. To safeguard remaining wild germplasm resources, future conservation efforts must prioritize implementing stricter protective measures to mitigate human disturbance and ensure long-term species persistence.

Future suitable habitat for *L. suprareticulata* under global warming scenarios reflects shifts in its potential distribution. Under SSP126, modest increases in temperature and precipitation are projected to expand its suitable habitat. In contrast, SSP245 and SSP585 are expected to induce substantial warming, reducing species adaptability and leading to a contraction of its future range—a pattern consistent with observations in other plant species [55,56]. However, this study does not account for future changes in soil conditions, which may further exacerbate habitat loss as soil characteristics evolve over the coming decades. To enhance predictive accuracy, future research should incorporate additional soil data to refine habitat suitability models for *L. suprareticulata*.

Karst limestone exhibits unique environmental characteristics [3], imposing selective pressures that drive the evolution of specialized plant adaptation. This study identified several adaptive genes within selective sweep regions of *L. suprareticulata* that contribute to its survival under these challenging environmental conditions. Notably, *Hsp101* and *CLPB3* enhance drought resistance [57,58,59], potentially buffering the species against rapid temperature fluctuations in limestone habitats. Additionally, *XI-I* contributes to nuclear elongation in various epidermal cell types [60], while *PDS3*—a critical enzyme in carotenoid synthesis—indirectly regulates photosynthetic efficiency and development processes [61]. Furthermore, *CYS-C1* improves resistance to pathogens and salt stress [62], and *TOPP4* regulates the photoreaction and Gibberellin (GA) signaling pathways to balance growth and defense [63,64,65].These findings provide critical insights into the molecular basis of plant adaptation to the karst limestone environment.

Temperature and precipitation are key drivers of genetic diversity in plants [66,67], as evidenced by their high environmental importance rankings in the GF model for *L. suprareticulata*. Using two GEA methods, we identified adaptive loci associated with temperature and precipitation. Annotated genes at these loci are involved in drought and salt stress responses, growth and development regulation, secondary metabolism, and defense mechanisms. For instance, *RabE1c* enhances plant drought resistance [68], while *PAL, SCL331*, and *CAD5* contribute to disease resistance [69,70]. Furthermore, *VAMP727*, *ABCB25*, and *UVH6* regulate growth and development, enabling plants to cope with environmental fluctuations [71,72,73]. Notably, *AtPIP1;4*, encoding a water channel protein, enhances drought tolerance by facilitating efficient transmembrane water transport [74,75]. Collectively, these genes enable *L. suprareticulata* to adapt to limestone karst environments and rapidly fluctuating climatic conditions through coordinated physiological responses.

The RONA values for *L. suprareticulata* were analyzed using five bioclimatic variables after addressing multicollinearity, under two shared socioeconomic pathways (SSP126 and SSP585). The four populations exhibited distinct adaptive responses to temperature and precipitation variables. Notably, bio16 exerted the strongest selection pressures on *L. suprareticulata* by reducing climatic suitability, thereby posing a greater maladaptive risk than other variables. Under future climate projections, Guangxi is anticipated to experience rising temperatures and increasingly erratic precipitation patterns. These changes, coupled with the region’s fragile karst ecosystems, may compromise plant resilience to extreme rainfall events [50]. Intense rainfall could also exacerbate soil erosion, further depleting the already scarce soil resources on limestone mountains [76]. Given the accelerating pace of climate change, the adaptive capacity of allelic variations may lag behind environmental shifts [77,78], potentially jeopardizing the long-term persistence of *L. suprareticulata*. Compounding these challenges, human activities are intensifying habitat fragmentation in limestone karst [30], thereby restricting gene flow and elevating extinction risk. To mitigate these threats, protective measures are urgently needed, including designating four populations as independent conservation units with tailored management strategies and implementing regular monitoring of population dynamics and habitat changes. Such efforts are critical for ensuring the long-term viability of this species.

## 4. Materials and Methods

### 4.1. Sampling, Resequencing, and SNP Calling

A total of 36 *L. suprareticulata* samples were collected from four distinct sites in Guangxi Zhuang Autonomous Region. (Figure 1a, Table 4). To ensure independence, individual plants were spaced at least 20 m apart. Fresh, tender leaves were harvested from each plant, and detailed geographical information (latitude, longitude, elevation) was recorded for each site. Samples were immediately divided into labeled packages, desiccated in silica gel (Sangon Biotech, Shanghai, China), and stored to maintain DNA integrity.

DNA was extracted from leaves using a modified CTAB method [79] and quality-assessed by agarose gel electrophoresis. High-quality DNA samples underwent library preparation, including fragmentation, size selection, end repair, adapter ligation, and amplification. Following library quality control, paired-end 150 bp (PE150) resequencing was performed on the DNBSEQ-T7 platform (BGI, Shenzhen, China). Raw reads were filtered using SOAPnuke v2.2.6 [80] with parameters ‘−n 0.001 −l 10 −q 0.5 −Q 2’, and the output quality score system was set to Phred+33, yielding high-quality clean reads.

The reference genome of *Lagerstroemia indica* [40] was obtained from the National Genomics Data Center (NGDC, https://ngdc.cncb.ac.cn/, accessed on 5 March 2025) and used for read mapping. The reference genome was indexed with BWA v0.7.17 [81], and paired-end reads were aligned to it using BWA-MEM, generating SAM files. The resulting SAM files were subsequently converted to BAM format, sorted, and processed for duplicate read marking using SAMtools v1.7 [82] and Picard v1.7 (https://broadinstitute.github.io/picard/, accessed on 10 March 2025), respectively. Variant calling was performed with BCFtools v1.9 [83] using the mpileup command, followed by preliminary filtering with VCFtools v0.1.16 [84]. The resulting VCF files were converted to PLINK-format binary files using PLINK v1.9 [85], excluding SNPs with low minor allele frequency (MAF) (<5%), high missing-data rate (>20%), or in high linkage disequilibrium (LD) (r^2^ < 0.2). After completing these steps, the final VCF file containing high-quality SNPs was available for subsequent analysis.

### 4.2. Population Structure and Demographic History Analyses

To elucidate the population structure of *L. suprareticulata*, we employed a comprehensive analytical framework integrating multiple methodological approaches. PCA was performed using the binary PLINK bed file, and the resulting population structure was visualized in Origin v2024 (OriginLab Corporation, Northampton, MA, USA). The VCF file was converted to FASTA format using the vcf2fasta package (https://github.com/santiagosnchez/vcf2fasta, accessed on 1 April 2025). Subsequently, a neighbor-joining (NJ) tree was constructed in MEGA v11 [86] using *L. indica* as an outgroup. Population structure was further examined using ADMIXTURE v1.3.0 [87], testing hypothetical ancestral populations (K) from 1 to 5, with the optimal K determined by minimizing CV error. Genetic diversity metrics—including observed heterozygosity (H_O_), expected heterozygosity (H_E_), π, and F_ST_—were calculated using VCFtools. Subsequently, site-specific population-scaled mutation rates (θ) were estimated from the BAM files using ANGSD v0.938 [88]. Tajima’s D was then calculated in sliding windows of 50 kb with a 10 kb step size, incorporating these θ estimates.

Mantel test from the vegan v2.7.1 R package (https://CRAN.R-project.org/package=vegan, accessed on 10 April 2025) was employed to assess relationships among geographic, environmental, and genetic distance matrices. Geographic distance was computed from latitude and longitude coordinates using the geosphere v1.5 R package (https://www.rdocumentation.org/packages/geosphere/versions/1.5-20, accessed on 12 May 2025). We downloaded 19 bioclimatic variables from WorldClim v2.1 (http://www.worldclim.org/, accessed on 14 May 2025), extracted their values at each sampling site, and constructed an environmental Euclidean distance matrix. Genetic distances were computed as F_ST_/(1 − F_ST_). To infer historical population dynamics, we employed SMC++ v1.15.2 [89], a program that utilizes a Sequentially Markov Coalescent (SMC) model to estimate the history of Ne from whole-genome sequence data. The analysis involved converting the VCF file into the SMC++ input format using the vcf2smc subcommand (https://github.com/popgenmethods/smcpp, accessed on 18 May 2025), with key parameters set to a mutation rate of 6.15 × 10^−9^ and a generation time of 4 years [40]. The resulting inference revealed the trajectories of Ne for the four populations.

### 4.3. Species Distribution Modeling

Occurrence data for *L. suprareticulata* were compiled from field surveys and verified records from three databases: China National Specimen Information Infrastructure (NSII, http://www.nsii.org.cn/, accessed on 20 May 2025), Chinese Virtual Herbarium (CVH, http://www.cvh.ac.cn/, accessed on 20 May 2025), and the Global Biodiversity Information Facility (GBIF Occurrence Download. Available online: https://www.gbif.org/occurrence/search?taxon_key=3988268, accessed on 20 May 2025). To ensure spatial independence, duplicate records were removed using ENMtools v2.0 [90], yielding 13 geographically distinct occurrence points for analysis. Environmental variables were sourced from multiple datasets: 19 climatic variables and three topographic parameters (elevation, slope and aspect) were obtained from WorldClim (http://www.worldclim.org/, accessed on 22 May 2025), while 11 soil-related variables were extracted from the Harmonized World Soil Database (HWSD) v1.2 (http://www.fao.org, accessed on 23 May 2025) (Table S2). Among these datasets, future climate projections were derived from the CMIP6’s high-resolution BCC-CSM2-MR model, which is optimized to simulate tropospheric air temperature and circulation patterns at both global and East Asian scales [91]. Paleoclimate data were generated using the CCSM4 model with WorldClim v1.4 (https://www.worldclim.org/data/v1.4/paleo1.4.html, accessed on 25 May 2025) at a 2.5 min spatial resolution. All environmental variables were extracted for the administrative regions in China using ArcGIS v10.8 (ESRI; https://www.esri.com/en-us/arcgis/products/index, accessed on 15 June 2025) and exported to ASCII format for subsequent analysis.

We performed species distribution modeling using MaxEnt v3.4.4 [92]. Occurrence records were randomly partitioned into 75% training and 25% test subsets. A fixed random seed was employed, and replicated runs were conducted using the bootstrap method. All other parameters were retained at their default settings. The final model output was derived by averaging predictions across ten replicated runs. To assess the relative importance of environmental predictors, we conducted a Jackknife test. Pearson correlation coefficients between predictors were calculated using R v4.1.1 (R Core Team, 2021) to mitigate overfitting, and the correlation matrix was visualized with the corrplot v0.95 (https://github.com/taiyun/corrplot, accessed on 20 June 2025) package. Among highly correlated pairs (|r| ≥ 0.85), we retained only the predictor with the higher contribution value.

Species distribution modeling was conducted across three time periods: (1) paleoclimatic, encompassing the LGM and the MH; (2) current, representing the period 1970–2000; and (3) future climate scenarios, including two periods: 2050 (2041–2060) and 2090 (2081–2100), under SSP126, SSP245, and SSP585. Soil and topographic variables were maintained constantly across both past and future conditions. Model performance was evaluated using the AUC metric and validated against the observed current distribution. The simulation results were imported into ArcGIS v10.8 (Esri, Redlands, CA, USA), where continuous values were reclassified into distinct suitable habitat categories, and the area of each category was quantified.

### 4.4. Selective Sweeps Analysis

RAiSD v2.9 [93] was used to identify positively selected loci by calculating the μ statistic, which integrates multiple signatures of a selective sweep and SNP vectors. First, the VCF file was partitioned by chromosome using the view command in BCFtools. Subsequently, μ statistics were computed with a window size of 50 SNPs, and the top 5% of μ values were selected to pinpoint loci under positive selection [93].

### 4.5. Genotype–Environment Association (GEA) Analyses

We employed two distinct approaches to identify adaptive SNPs associated with bioclimatic variables at the genome-wide scale. Prior to GEA analyses, the relative importance of environmental variables was assessed using a GF model implemented in the GradientForest v0.1.37 R package [94], with the ntree parameter set to 500. For highly correlated pairs of bioclimatic variables (|r| ≥ 0.85), only the variable with higher explanatory power was retained for analysis.

A univariate LFMM, as implemented in the LEA v3.6.0 R package [95], was used to identify associations between allele frequencies and environmental variables. To account for population structure, k = 4 latent factors were specified based on ADMIXTURE-inferred ancestry clusters. RDA has been shown to perform better than other multivariate GEA approaches while maintaining low false positive rates [96]. RDA analyses were performed using the vegan v2.7.1R package to identify SNPs strongly associated with multivariate environmental axes. *p*-values were corrected for multiple testing using the qvalue R package (https://github.com/StoreyLab/qvalue, accessed on 12 August 2025) at a false discovery rate (FDR) threshold of 0.01.

Consensus environmentally adaptive SNPs identified by both GEA methods were retained for subsequent analysis. These SNPs were extracted using VCFtools, and flanking genes were identified through comparison with the reference genome. Protein sequences were annotated using eggnog-mapper, followed by GO enrichment analysis with GOWINDA v1.12 [97] and KEGG enrichment analysis with TBtools-II v2.363 [98]. BLAST [99] was then used to align annotated gene sequences against the NCBI database, revealing genes potentially involved in the environmental adaptation of *L. suprareticulata*.

### 4.6. Risk of Non-Adaptedness (RONA) Under Future Climate Scenarios

RONA quantifies the average allele frequency shift in environmentally linked SNPs under future climate scenarios, reflecting potential maladaptation risks [100]. RONA was assessed for *L. suprareticulata* populations using pyRONA [100], which integrates current and future allele frequency data with bioclimatic variables to quantify maladaptation risks under climate change. Future climate projections were based on two Shared Socioeconomic Pathways: a low-emission scenario (SSP126) and a high-emission scenario (SSP585). Six bioclimatic variables aligned with prior GEA analyses and allele frequencies of adaptive SNPs identified via LFMM were incorporated to assess adaptive capacity.

## 5. Conclusions

This study utilized genomic data to investigate the genetic diversity, population structure, and demographic history of *L. suprareticulata*. Geographic isolation has resulted in pronounced genetic differentiation among its populations. Demographic analysis revealed an initial population expansion followed by sustained contraction in effective population size, underscoring the need for ongoing monitoring of demographic trends. By integrating environmental and genomic data, we modeled the suitable habitats of *L. suprareticulata* and predicted a future contraction of high-suitability areas, with the WHS population particularly vulnerable. Selective sweep and GEA analyses identified genomic regions containing key genes associated with adaptation to limestone karst environments. Additionally, RONA assessments quantified future survival risks for *L. suprareticulata* under varying climate scenarios. Collectively, these findings provide insights into the environmental adaptation mechanisms of *L. suprareticulata* in the limestone karst ecosystem. Future conservation strategies should incorporate these findings to effectively safeguard the wild germplasm resources of *L. suprareticulata* within these vulnerable landscapes.

## Figures and Tables

**Table 1:** Summary of genetic diversity parameters for four *L. suprareticulata* populations: observed heterozygosity (H_O_), expected heterozygosity (H_E_), inbreeding coefficient (F_IS_), nucleotide diversity (π), and Tajima’s D.

	H_O_	H_E_	F_IS_	π	Tajima’s D
BL	0.3796	0.3242	0.1709	0.002116	0.1225
JCS	0.4595	0.3843	0.1958	0.002113	0.1480
LHS	0.4009	0.3376	0.1876	0.002137	0.1319
WHS	0.7211	0.5814	0.2402	0.001779	0.4493

**Table 2:** Percent contribution and permutation importance of seven most influential variables in MaxEnt model for *Lagerstroemia suprareticulata* habitat suitability.

Variable	Percent Contribution (%)	Permutation Importance (%)
bio6	38.6	52.4
awc_class	22.3	3.7
bio1	14.5	28.3
t_clay	13.4	2.1
s_ph_h2o	5.9	1.1
bio9	4.3	3
bio10	1.1	9.4

**Table 3:** Potential suitable areas (km^2^) for *Lagerstroemia suprareticulat* under past (Last Glacial Maximum, Mid-Holocene), present, and future climate scenarios (SSP126, SSP245 and SSP585). Values in parentheses represent percentage change relative to the current baseline.

Period	Lowly Suitability Area (km^2^)	Moderately Suitable Area (km^2^)	Highly Suitable Area (km^2^)	Total Suitable Area (km^2^)
Current	62,752.78	22,761.11	11,613.89	96,127.78
Last Glacial Maximum	87,378.47 (139.24%)	27,118.06 (119.14%)	21,388.89 (184.17%)	135,885.42 (141.35%)
Mid-Holocene	97,395.83 (155.21%)	39,930.56 (165.43%)	28,229.17 (243.06%)	165,555.56 (172.22%)
SSP126-2050	15,708.33 (25.03%)	10,240.97 (44.99%)	12,570.83 (108.23%)	38,520.13 (40.07%)
SSP126-2090	25,222.22 (40.19%)	15,551.39 (68.32%)	16,470.83 (141.82%)	57,244.44 (59.55%)
SSP245-2050	63,350.01 (100.95%)	32,315.28 (141.98%)	18,599.31 (160.15%)	114,264.6 (118.87%)
SSP245-2090	39,619.44 (63.14%)	14,392.36 (63.23%)	14,437.5 (124.31%)	68,449.3 (71.21%)
SSP585-2050	46,157.64 (73.55%)	18,386.11 (80.78%)	20,657.64 (177.87%)	85,201.39 (88.63%)
SSP585-2090	27,765.97 (44.25%)	9647.22 (42.38%)	12,985.42 (111.81%)	50,389.61 (52.42%)

**Table 4:** Geographical information of sampling locations of *Lagerstroemia suprareticulata*.

Population	Sampling Location	Longitude (°)	Latitude (°)	Elevation (m)	Individuals
BL	Bangliang Gibbon National Nature Reserve, Jingxi City	106.4989	22.9164	754	16
JCS	Chongzuo White-headed Langur National Nature Reserve, Jiuchongshan, Chongzuo City	107.8656	22.4511	124	6
LHS	Longhushan Nature Reserve, Longan County	107.6141	22.9768	213	12
WHS	Wohushan, Liuzhou City	109.4104	24.2797	103	2

## Data Availability

The data that support the findings of this study have been deposited into the National Genomics Data Center (NGDC) with the accession number CRA035771.

## Supplementary Materials

The following supporting information can be downloaded at: https://www.mdpi.com/article/10.3390/plants15040629/s1, Figure S1: Pearson correlation coefficients among the 33 environmental predictors used in MaxEnt analysis; Figure S2: Jackknife analysis of regularized training gain for *Lagerstroemia suprareticulata* showing variable contributions to the MaxEnt model; Figure S3: Projected suitable habitats for *Lagerstroemia supraretic*ulata during the Last Glacial Maximum (LGM), Mid-Holocene (MH), and Current period, as predicted by MaxEnt modeling; Figure S4: Pearson correlation matrix of 19 bioclimatic variables; Figure S5: Variable importance from gradient forest (GF) analyses, quantified as the R^2^-weighted contribution, reflecting the explanatory power of each variable in the model. Table S1: Pairwise F_ST_ values among four populations; Table S2: List of all environment variables used in MaxEnt, including their full names; Table S3: GO enrichment analysis of the regions under positive selection; Table S4: KEGG enrichment analysis of the regions under positive selection; Table S5: GO enrichment analysis of the high-confidence adaptive SNPs; Table S6: KEGG enrichment analysis of the high-confidence adaptive SNPs.

## Abbreviations

The following abbreviations are used in this manuscript:

AUC	Area under the receiver operating characteristic curve
BLAST	Basic Local Alignment Search Tool
CV	Cross-validation
CVH	Chinese Virtual Herbarium
EN	Endangered
FDR	False Discovery Rate
GA	Gibberellin
GEA	Genotype–environment association
GF	Gradient Forest
GO	Gene Ontology
HWSD	Harmonized World Soil Database
KEGG	Kyoto Encyclopedia of Genes and Genomes
LD	Linkage disequilibrium
LFMM	Latent Factor Mixed Model
LGM	Last Glacial Maximum
MAF	Minor allele frequency
MaxEnt	Maximum entropy modeling
MH	Mid-Holocene
NCBI	National Center for Biotechnology Information
NGDC	National Genomics Data Center
NJ	Neighbor-joining
Ne	Effective population size
PCA	Principal component analysis
RDA	Redundancy analysis
RONA	Risk of non-adaptedness
SDM	Species distribution model
SNP	Single-nucleotide polymorphism
SSP	Shared Socioeconomic Pathway
VCF	Variant Call Format
