# Supplementary material for: Genomic Insights into Adaptation of Lagerstroemia suprareticulata to Limestone Karst Habitats
Source: Plants (Basel). 2026 Feb 16;15(4):629. doi: 10.3390/plants15040629 (PMC12944261; doi:10.3390/plants15040629)
Supplement: Supplementary file 1 [file plants-15-00629-s001.zip › Supplementary.pdf]

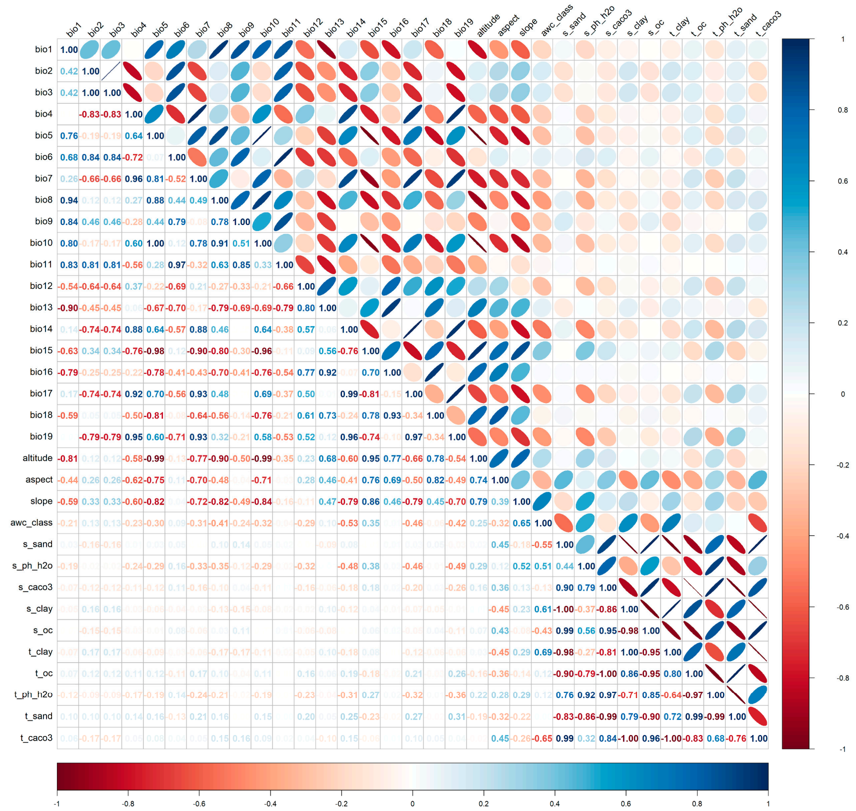

Figure S1. Pearson correlation coefficients among the 33 environmental predictors used in MaxEnt analysis.

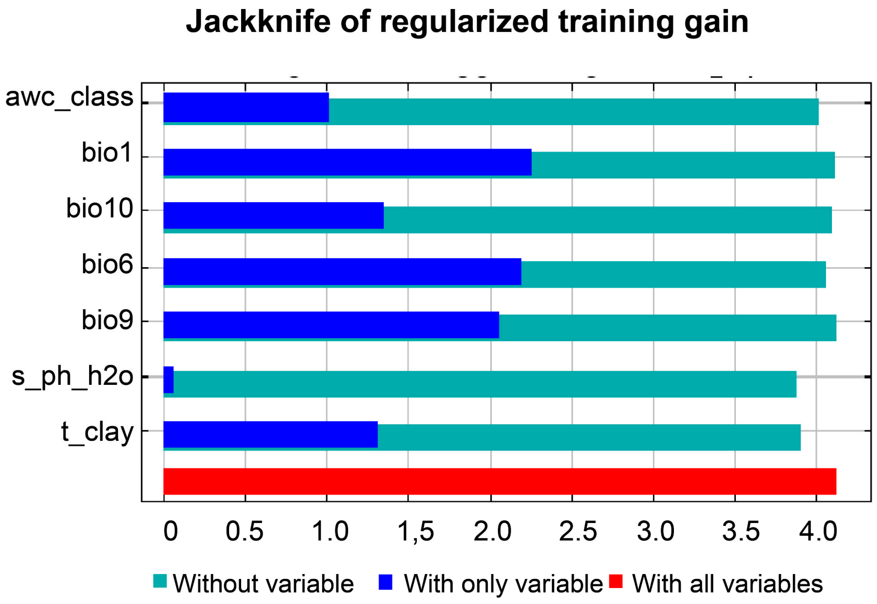

Figure S2. Jackknife analysis of regularized training gain for *Lagerstroemia suprareticulata* showing variable contributions to the MaxEnt model.

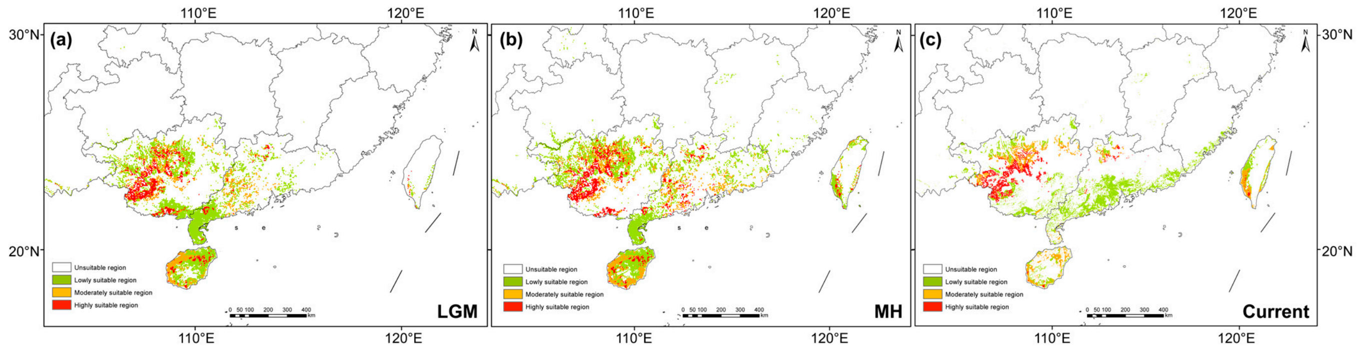

Figure S3. Projected suitable habitats for *Lagerstroemia suprareticulata* during the Last Glacial Maximum (LGM), Mid-Holocene (MH), and Current period, as predicted by MaxEnt modeling.

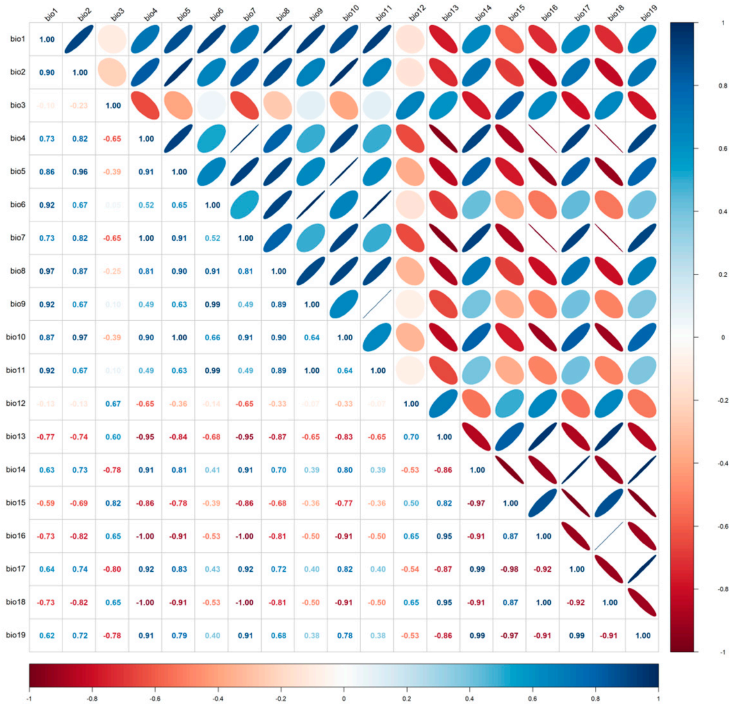

Figure S4. Pearson correlation matrix of 19 bioclimatic variables.

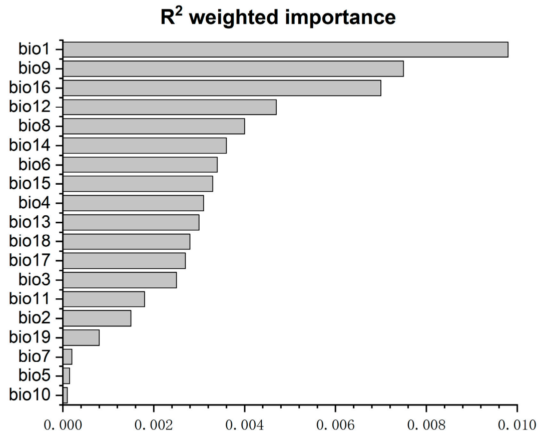

Figure S5. Variable importance from gradient forest (GF) analyses, quantified as the R2-weighted contribution, reflecting the explanatory power of each variable in the model.
